# Alanine aminotransferase assay biosensor platform using silicon nanowire field effect transistors

**DOI:** 10.1038/s44172-023-00057-4

**Published:** 2023-03-01

**Authors:** Katherine A. Muratore, Dan Zhou, Jiangang J. Du, John S. Chlystek, Kasra Motesadi, Erik K. Larsen, Brenda M. Molgora, Tetz C. Lee, Sudhakar Pamarti, Shyamsunder Erramilli, Pritiraj Mohanty

**Affiliations:** 1 FemtoDx, Inc., 8484 Wilshire Blvd, Suite 630, Beverly Hills, CA 90211 USA; 2 Dept. of Electrical and Computer Engineering, University of California, Los Angeles, CA 90095 USA; 3 Dept. of Physics, Boston University, 590 Commonwealth Avenue, Boston, MA 02215 USA

**Keywords:** Biosensors, Proteins, Assay systems

## Abstract

Frequent monitoring of serum alanine aminotransferase (ALT) activity is essential to prevent drug-induced liver injury (DILI). Current ALT assays are restricted to centralized clinical laboratories, making frequent patient monitoring logistically difficult. To address this, we demonstrated the capability of commercial foundry manufactured silicon nanowire field effect transistor (SiNW-FET) biosensors in a form factor that enables frequent near-patient monitoring. Here, we designed an ALT assay, by coupling the ALT-catalyzed production of pyruvate to the reduction of ferricyanide, enabling both spectrophotometric and electrical measurement of ALT activity. The two methods yield comparable ALT activity detection across a dynamic range wide enough to monitor patients at risk for DILI. This study demonstrates kinetic activity measurement of an endogenous enzyme using uncoupled SiNW-FETs, and commercial manufacturing of SiNW-FET sensor arrays for use in a portable biosensor platform.

## Abstract (web summary)

Alanine aminotransferase (ALT) activity is an important indicator to determine drug induced liver injury. However, its measurement is limited to centralized laboratories. Muratore, Zhou, and colleagues designed an ALT assay biosensor platform using silicon nanowire field effect transistor arrays. The biosensor could monitor ALT enzyme activity electrically, with comparable precision to spectrophotometric detection. This biosensor platform will enable routine liver function monitoring in settings both inside and outside of the clinic.

## Introduction

Liver toxicity is the most common cause for premature termination of drug trials and removal of previously approved drugs from the market^1,2^. Therefore, drug-induced liver injury (DILI) is a major safety concern throughout the drug development pipeline^3^. DILI events traditionally fell into two categories: direct hepatoxicity and idiosyncratic hepatotoxicity^4^. The latency period for direct DILI is typically 1–5 days after dosage, while idiosyncratic DILI is not dose dependent, and the latency period varies from days to years^5^. DILI resulting from the action of a drug, rather than the drug itself, has recently been characterized as indirect hepatoxicity and typically takes months to manifest^5^. The diversity in DILI latency period must be addressed with frequent patient monitoring.

The first line screening for acute liver injury is to assay serum activity of the enzyme alanine aminotransferase (ALT, BRENDA: EC 2.6.1.2)^6^. As ALT is present in high concentrations in hepatocytes and released upon liver injury, its activity determination is the preferred test for ongoing monitoring of DILI^7^. There are five defined severity levels of DILI, Level 1–Level 5, which correlate with elevated serum ALT activity^8^. When serum ALT activity increases above 3X the upper limit of normal (ULN) during a clinical trial (Level 2 DILI), it is recommended to assay the patient’s ALT level every three days^9,10^. If serum ALT activity remains above 5X ULN for two weeks or exceeds 8X ULN at any point (Level 3 DILI), the U.S. Food and Drug Administration (FDA) requires removing the patient from the clinical trial^2,7^. The severity of DILI experienced in a clinical trial may vary by the drug and the population exposed to it^8^, therefore any method for monitoring ALT activity must have a dynamic range that covers up to 8X the ULN, encompassing both Level 2 and Level 3 DILI. The current gold-standard ALT activity measurement is an absorbance-based assay that requires a large, automated platform in a clinical laboratory. With ALT measurements limited to a centralized laboratory, delays between prognostic indications of liver failure and subsequent clinical intervention are likely unavoidable. In addition to logistical burden and laborious process, these tests are further impractical in resource limited settings due to large instrument form factor, acquisition costs, and operator requirements.

Silicon nanowire field effect transistors (SiNW-FETs) that can measure the change in ion concentration in an electrolyte solution, are a promising solution to address the above limitations. SiNW-FETs have been widely employed to detect numerous physiologically relevant charged species^11–14^, including protein^15–19^ and DNA^20–22^ biomarkers. The nanowire architecture boosts the sensitivity of a FET several orders of magnitude^12,23^ which is largely achieved by minimizing the footprint of sensing elements so that they approach the dimension of the biomolecules to be monitored. The sensitivity of sensors is thus greatly enhanced due to the increased surface area to volume ratio^12,24,25^. Specificity for label-free detection of individual biomarkers is typically achieved by functionalization of the sensor surface with analyte binding partners^15–22^ or ion sensitive functional groups^11–14,24^. As enzyme activity is measured by the rate of its catalyzed reaction, real-time data recording by SiNW-FET allows the determination of reaction kinetics^15^, and provides an opportunity to develop diagnostic assays for clinically relevant biomarker enzymes using SiNW-FETs. Enzyme coupled FETs have been widely used to monitor the conversion of a specific substrate^24,26–29^ but to our knowledge, an uncoupled SiNW-FET has not been used to kinetically monitor the activity of an endogenous human enzyme.

In this work, we combined the principle of FETs^30,31^ and SiNW biosensing architecture to obtain the needed sensing capability for serum ALT enzyme activity on a portable SiNW-FET biosensor system. With product development and commercialization in mind, our SiNW-FET biosensors were designed and fabricated by scalable and wafer-level manufacturing processes. Hundreds of nanowire sensor dies were simultaneously produced. The sensing surfaces were modified to facilitate the measurement of serum ALT activity using an assay chemistry optimized for SiNW-FET detection. These sensors in a portable, low-cost device that can detect abnormally elevated ALT levels near patient will enable frequent monitoring of DILI in clinical trial participants to prevent severe liver damage. This is not otherwise possible for outpatients in traditional clinical settings. We sought to address this unmet clinical need by generating and characterizing a commercially scalable SiNW-FET biosensor platform, capable of detecting a wide dynamic range of serum ALT activity.

## Results

### SiNW-FETs as portable ALT biosensors

Frequent monitoring of patient serum ALT activity is required during pharmaceutical clinical trials to determine the occurrence and severity of DILI. A reliable, portable, and inexpensive biosensor that can be used outside a laboratory setting would provide an easy option for monitoring of ALT during drug treatment protocols. For example, over a typical treatment period of 3-6 months with an immunocheckpoint inhibitor, it is recommended to initially monitor ALT weekly, increasing to every 3 days if ALT is elevated beyond 3X the patient’s upper limit of normal^10^. We fabricated SiNW-FET biosensors for this purpose and for eventual integration into a portable biosensor platform. Leveraging standard semiconductor processing in commercial foundries is crucial to keep the cost of SiNW-FET biosensors low, making it affordable and available for a broad population of patients.

**Fig. 1:**
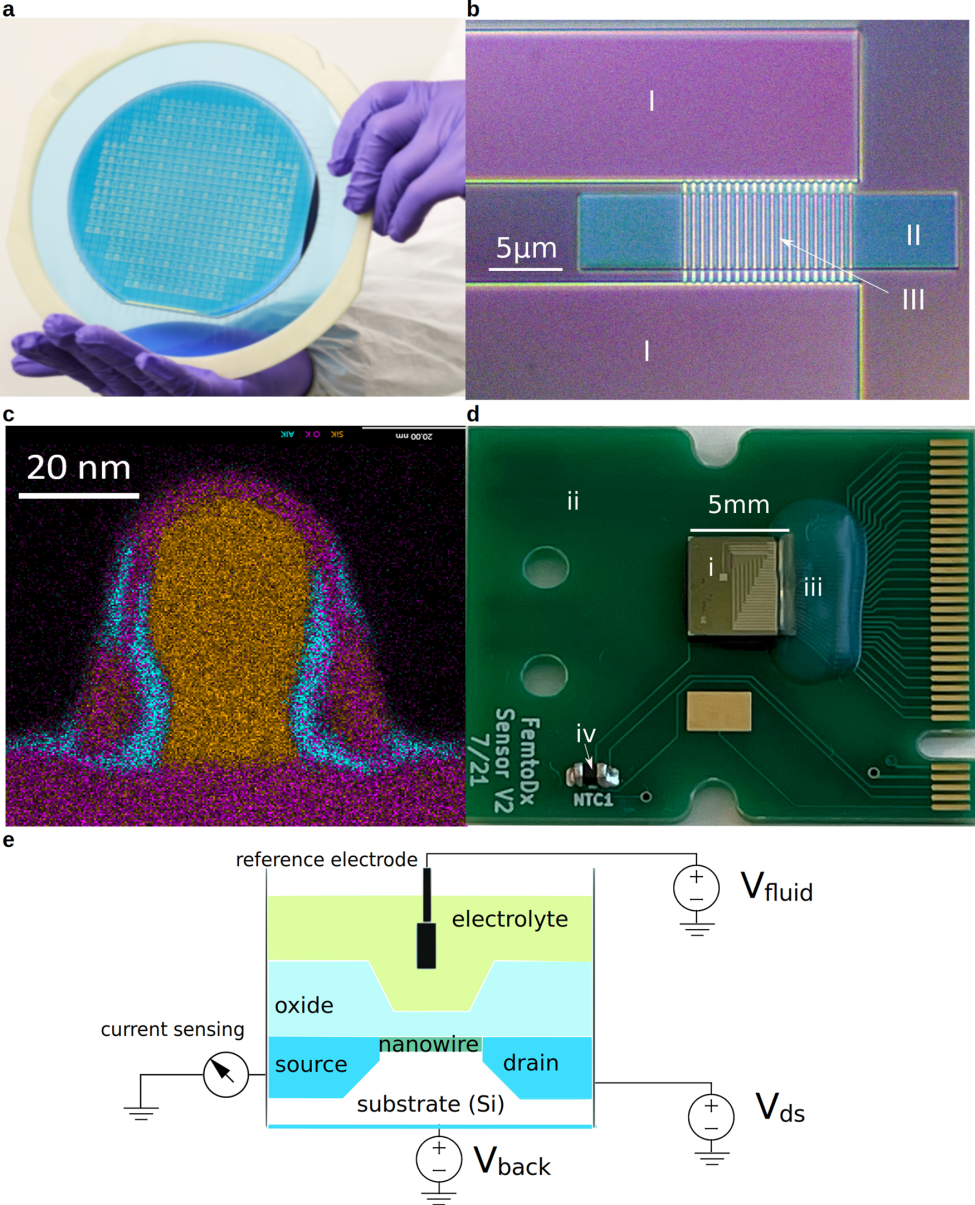
Nanowire sensors and ALT measurement by electrical current sensing. **a** An 8-inch silicon wafer carrying up to 700 sensor dies that each has multiple nanowire sensor arrays by wafer-level manufacturing processes. **b** Optical microscopic image showing the structure of a nanowire sensor that is comprised of drain/source terminals (I), a sensing area access slit (II), and a nanowire array (III). **c** High-resolution scanning transmission electron microscopy (TEM) image showing the cross-section of a nanowire, indicating a nanowire width of ~20 nm and the thin dielectric layer. **d** Optical image of a wire-bonded die (i) on a printed circuit board (PCB; ii) where it carries the sensor die with encapsulated bonding wires (iii) and an on-board thermometer(iv). **e** System diagram illustrating the principle of operation of using a nanowire sensor to detect ALT enzyme activity.

Silicon nanowire sensor arrays were fabricated using a wafer-level manufacturing process originated and scaled up from research laboratory protocols^11,32^. The process involves silicon-on-insulator (SOI) wafers as a substrate for hosting SiNW-FET biosensor dies. Commercially available SOI wafers (SOITEC, Bernin, France) produced by mature, industrial grade processes carry a single crystalline silicon epitaxy layer. This layer has minimal crystalline defects and enables the reliable operation of nanowire sensors. Identical design patterns of nanowire sensors were transferred to the SOI substrate in the unit of a die via planar layer-by-layer semiconductor processing techniques (Fig. 1a). Approximately 700 sensor dies were produced from an 8-inch diameter wafer. The key elements of each nanowire sensor die included the drain and source terminals, nanowire array, and sensing area (Fig. 1b). A cross-sectional transmission electron microscopy (TEM) image revealed nanowire width as narrow as ~20 nm (Fig. 1c). Each sensor die is then diced off from the wafer and surface-functionalized with 3-aminopropyl-triethoxysilane (APTES) for ALT detection. To facilitate electrical readout using a multiplexing platform, the sensor die is mounted and electrically connected to a predesigned printed circuit board (PCB) via commercial die-attachment and encapsulated wire-bonding services. With dimensions of 5.0 mm×5.0 mm, the sensor die is small enough to fit onto the PCB board 2.0 cm × 2.4 cm in size (Fig. 1d).

While arranged as a nanowire sensor array consisting of 7 test sensors on each die, every individual test sensor is electrically accessible via the finger pads located at the right end of the PCB board (Fig. 1d). From there, the signal from each sensor is recorded on a multiplexing platform. Each sensor operates independently on the principle of SiNW-FET. That is, each nanowire bridging the drain and source terminal forms an electrical channel with a finite electrical conductivity. When the sensing surfaces of the nanowire sensor are exposed to an electrolyte solution, the presence of biomarkers changes the surface ionic charge density along the liquid/sensor interface and modulates the conductivity of the nanowire channel through a field effect. The change in conductivity is detectable using a sensitive ammeter under a fixed voltage difference across the source and drain terminals, gated by additional potentials applied to the silicon substrate (back-gate voltage) and to a reference electrode in the solution (fluid-gate voltage). A set of dependable fluid-gate and back-gate voltages helps maintain a favorable signal-to-noise ratio and a consistent baseline conductivity of the nanowire channel (Fig. 1e). As our assay was performed in physiological conditions in the presence of salts, we estimate the Debye screening length to be ~0.7 nm. Therefore, due to the short Debye screening length, the response of each sensor to our assay is local. This is possible because the separation between each sensor and each array is greater than the estimated Debye screening length. As a result, concurrent measurements of ALT signals from multiple sensors are viable without the concern of interdependence.

### Application of ferricyanide–ferrocyanide redox couple to ALT assay

Absorbance based ALT assays on automated clinical platforms, in accordance with IFCC guidelines, measure the reduction rate of pyruvate by protonated (H+) nicotinamide adenine dinucleotide (NADH) in a reaction catalyzed by lactate dehydrogenase^33^. Conversion of NADH to nicotinamide adenine dinucleotide (NAD+) is monitored spectrophotometrically at 340 nm. To design a SiNW-FET compatible ALT assay, we sought to couple ALT generation of pyruvate to a change in charge. The changes in local ion concentration alter the surface potential and electric field, which modulate the resistance of SiNW-FETs^11^.

**Fig. 2:**
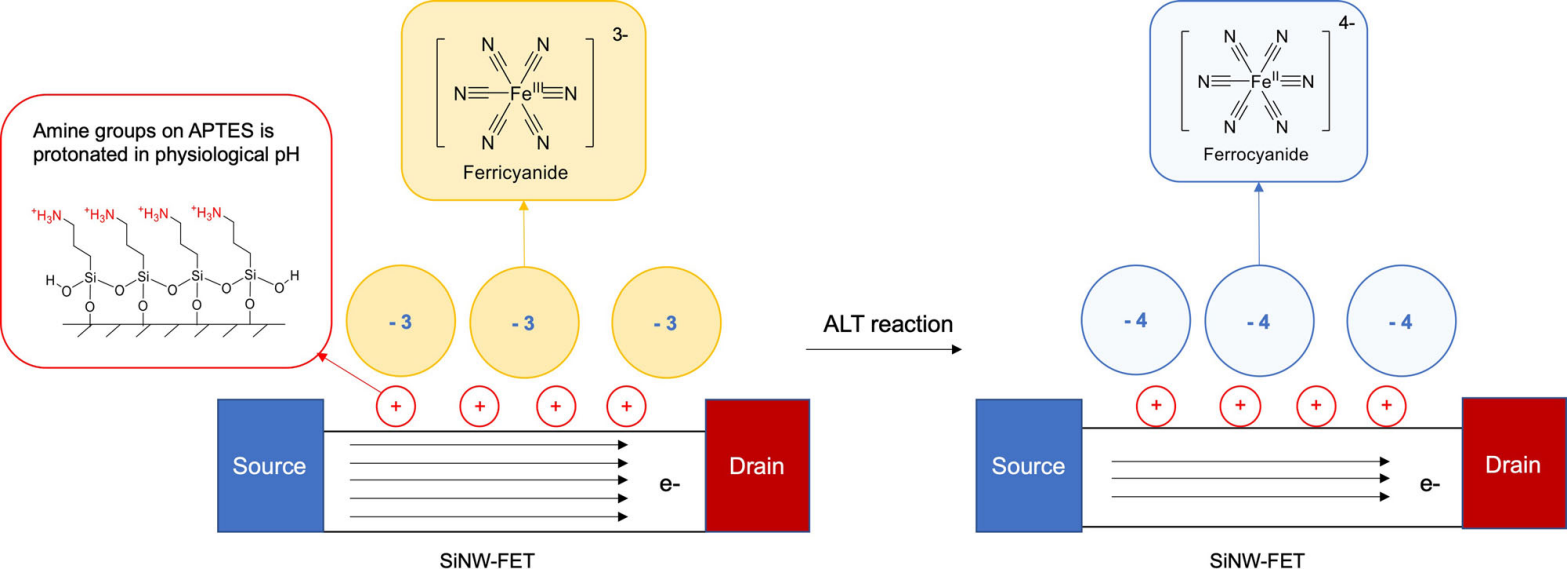
ALT assay is measured through redox reaction of ferricyanide-to-ferrocyanide on SINW-FET biosensors. ALT enzyme activity is detected by a change in local ion concentration to modulate the conductance of SiNW-FETs. The SiNW-FETs are functionalized with APTES, an aminosilane group that is protonated at physiological pH. This results in positively charged groups on the surface of the SiNW-FET, which attract negatively charged species and generate a field effect on sensors. In ALT assay, pyruvate produced by ALT-catalyzed reaction is coupled to pyruvate oxidase and reduces ferricyanide to ferrocyanide. As the number of electrons in the negatively charged ions changes increases, the surface charge in the vicinity of the SiNW-FET becomes more negative, leading to a measurable decrease in conductance for the type of sensors that use electron as its charge carrier.

Pyruvate oxidase (POX, BRENDA: EC 1.2.3.3), a flavoprotein, catalyzes the oxidative decarboxylation of pyruvate to yield acetate and carbon dioxide^34^. Electrochemical assays for ALT activity based on POX modified electrodes^35–37^ or employing ferrocene containing compounds as redox mediators^38–40^ have been reported previously. The final step in the reaction mechanism of POX in vivo is the reoxidation of the flavin by ubiquinone^41^. In vitro, ferricyanide is used as a soluble oxidant to monitor POX enzyme activity spectrophotometrically^42,43^. When used as an oxidant, ferricyanide is reduced to ferrocyanide, resulting in a change in local ion charges that can be detected by a SiNW-FET^24,26^. Primary amines from APTES layer functionalized on the surface of the sensor, are protonated at physiological pH in serum samples (Fig. 2). The negatively charged ferricyanide ions are attracted close to the sensor surface by electrostatic force. Reactions that convert ferricyanide to ferrocyanide lead to changes in electric field near the surface of the sensor, due to the difference in the ion charges. We have validated that APTES functionalization is essential to the sensitivity of detecting the conversion of the negatively charged ions (Supplementary Fig. 1), which allows this reported conjugation-free method, without coupling any enzyme or substrate on the sensor surface for enzyme activity measurement.

We designed our ALT assay by coupling the production of pyruvate to the reduction of ferricyanide though POX for optical and electrical detection. The designed ALT assay chemistry, conducted at 37 °C, begins with ALT converting L-alanine and alpha-ketoglutarate to pyruvate and glutamate. POX converts pyruvate to acetyl phosphate and reduces flavin adenine dinucleotide (FAD) to hydroquinone form of FAD (FADH_2_). Ferricyanide subsequently reduces to ferrocyanide via oxidation of FADH_2_. Although protons are also produced in the assay, serum has enough buffering capacity to yield a much smaller signal change due to pH compared to the ferricyanide to ferrocyanide conversion (Supplementary Fig. 2).

$${{{{{\rm{alpha}}}}}}{\mbox{-}}{{{{{\rm{ketoglutarate}}}}}}+{{{{{\rm{L}}}}}}{\mbox{-}}{{{{{\rm{alanine}}}}}}\mathop{\leftrightharpoons}^{{{{{\mathrm{ALT}}}}}} {{{{{\rm{glutamate}}}}}}+{{{{{\rm{pyruvate}}}}}}$$

$${{{{{\rm{pyruvate}}}}}}+{{{{{\rm{phosphate}}}}}}+{{{{{\rm{FAD}}}}}}\mathop{\leftrightharpoons }\limits_{{{{{{\rm{TPP}}}}}},{{{{{{\rm{MgCl}}}}}}}_{2}}^{{{{{\mathrm{POX}}}}}}{{{{{\rm{acetylphosphate}}}}}}+{{{{{{\rm{CO}}}}}}}_{2}+{{{{{{\rm{FADH}}}}}}}_{2}$$

$$2{{{{{\rm{Fe}}}}}}({{{{{\rm{CN}}}}}})_{6}^{3-} + {{{{{\rm{FADH}}}}}}_{2} \to 2{{{{{\rm{Fe}}}}}}({{{{{\rm{CN}}}}}})_{6}^{4-} + {{{{{\rm{FAD}}}}}}+2{{{{{\rm{H}}}}}}^{+}$$

### Analytical performance of the ALT assay

**Fig. 3:**
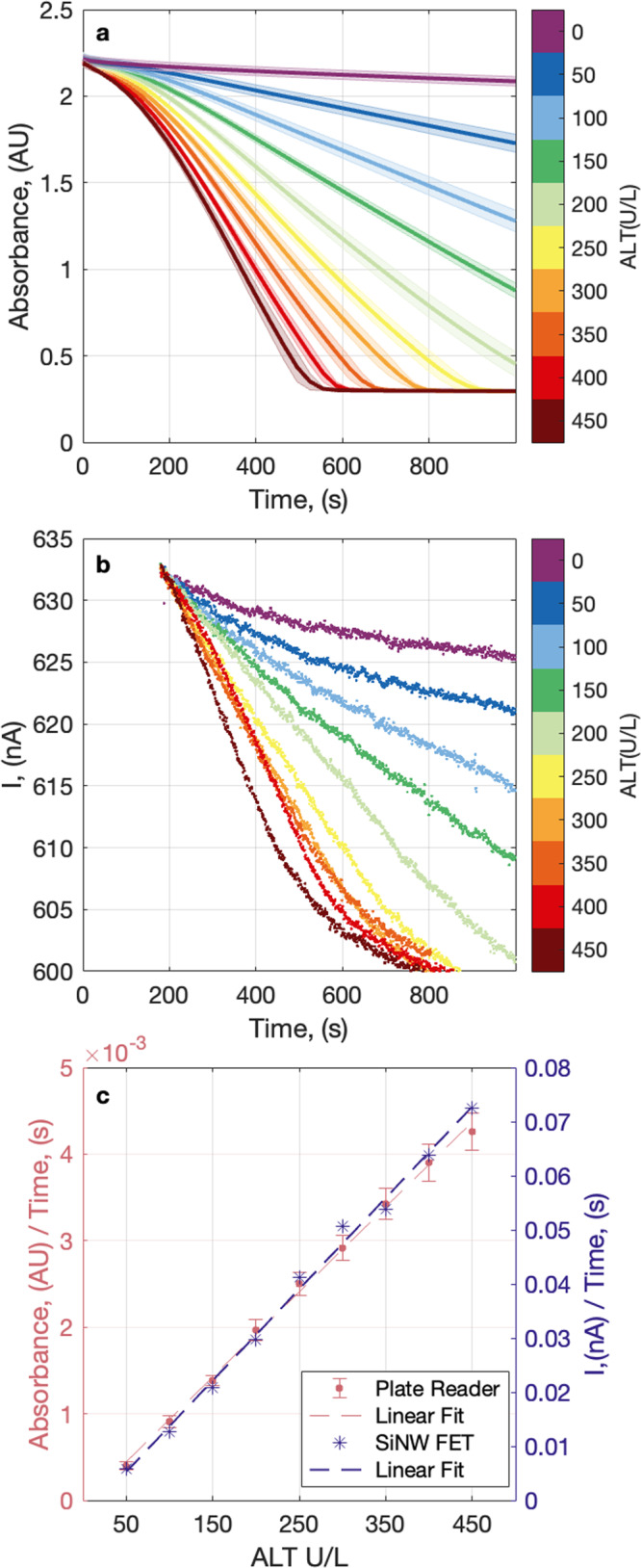
Ferricyanide reduction, as a readout of ALT assay, can be monitored both optically and electrically. The SiNW FET data in this figure (subplots **b** and **c**) are from a single experiment, repeated once. **a** Absorbance (AU) at 420 nm vs time (s) of a dilution series of ALT, 0–450 U/L using a microplate spectrophotometer. Each concentration is represented in a different color as indicated in the color bar, the center line is the mean, and the shaded area represents ± standard deviation of *n* = 9 assays (0–400 U/L ALT) or *n* = 6 assays (450 U/L ALT). Absorbance was measured at 30 s intervals. **b** Raw current (nA) vs time (s) of a dilution series of ALT, 0–450 U/L measured using a single silicon nanowire biosensor. Each concentration is represented in a different color as indicated in the color bar. Each curve is offset to an equal baseline at 180 s. **c** Standard curves from optical and electrical measurements show a linear trend between the signal and ALT activity. Rose colored data series is absorbance change rate in the time window 250–500s (AU/s) vs ALT (U/L). Values represent mean ± standard deviation of *n* = 9 assays (0–400 U/L ALT) or *n* = 6 assays (450 U/L ALT). Indigo colored data series is current change rate in the time window 250s-500s (nA/s) vs ALT (U/L).

The first focus of this report was to test the performance of the proposed chemistry to measure ALT enzyme kinetics over a clinically relevant linear range. Unless otherwise noted, the ALT enzyme standard was assayed in-house to determine activity (Supplementary Fig. 3). The assay chemistry was tested by adding a dilution series of ALT and assay reagent mixture to a 96-well microplate and monitoring the absorbance at 420 nm for 20 min at 37 °C. The ALT reaction was monitored kinetically by the consumption of ferricyanide during the assay period, reflected in a decrease in absorbance at 420 nm (Fig. 3a). The rate of the reaction is proportional to ALT activity, which is illustrated by the linear correlation between the slope of the ferricyanide consumption and ALT activity (Fig. 3c).

The upper limit of normal (ULN) for serum ALT activity varies at different laboratories, influenced by the clinical diversity in local reference populations^44^. To comply with FDA clinical trial stopping rules for DILI, any assay for ALT must cover up to 8X ULN (Supplementary Table 1)^2^. As the goal of our assay is to monitor serum ALT activity as an indicator for DILI, we sought to design the assay linear up to 8X ULN, with 50 units per liter (U/L) as 1X ULN to cover an inclusive range for most populations. To demonstrate the assay linear range, we constructed a standard curve of slope vs ALT activities and confirmed that the assay was linear between 50 and 450 U/L (Fig. 3c). The 95% confidence interval for the slope of linear regression line was [9.4e−6, 1.0e−5], AU/s vs ALT (U/L). *R*^2^ was 0.99 for the linear fit of the calibration curve, indicating the assay was linear over the target ALT activity range of interest. The limit of blank (LOB), defined as the minimum distinguishable analytical signal, ${S}_{m}$, was then calculated^45,46^

1 $${S}_{m}={\underline{S}}_{bl}+3{s}_{bl}$$

here ${\underline{S}}_{bl}$ is the mean signal from *n* = 10 measurements of samples with 0 U/L ALT, 1.1e−4 ± 1.1e−5 AU/s, ${s}_{{bl}}$ is the standard deviation of the signal from *n* = 10 blank measurements, 4.4e–6 ± 4.4e−7 AU/s, and we obtained an ${S}_{m}$ equal to 1.2e−4 ± 1.2e−5 AU/s.

The slope from the calibration curve in Fig. 3c, $m,$ was then used to calculate the limit of detection (LOD), ${c}_{m}$, defined as the smallest quantity of analyte that is statistically significant from a blank sample^45,46^, as follows:

2 $${c}_{m}=\frac{3s}{m}$$

here $s$ is the standard deviation from *n* = 10 measurements of 50 U/L ALT, 1.3e−5 ± 1.3e−4 AU/s, and we obtained a LOD equal to 4.1 ± 0.4 U/L ALT.

### ALT assay using SiNW-FET biosensors

The next focus of this report was to test the ALT assay chemistry we developed on the SiNW-FETs, which have been previously used to detect proteins^15–19^, nucleic acids^20–22^, and viruses^47,48^ but have not been used for an activity assay of an endogenous human enzyme. In this study, we used a sensor die with SiNW-FETs functionalized with primary amines to detect ALT enzyme activity in human serum. To perform the ALT assay on the SiNW-FETs, a dilution series of ALT samples were individually combined with assay reagent mixture and delivered to the sensor using a syringe and flow chamber. Throughout each ALT assay the direct current through the nanowire was recorded in real time on a SiNW-FET biosensor for 20 min (Fig. 3b). The electronic reading of the ALT assay monitors the consumption of ferricyanide during the assay period, reflected in a decrease in baseline current. The rate of decrease in baseline current is representative of the reaction rate (Fig. 3c). The 95% confidence interval for the slope of linear regression line was [1.6e−4, 1.8e−4], nA/s vs ALT (U/L). *R*^2^ was 0.99 for the linear fit of the calibration curve, indicating that the reaction rate was proportional to ALT activity and the assay is linear between 50 and 450 U/L ALT, comparable to sophisticated optical measurement using a microplate reader.

**Fig. 4:**
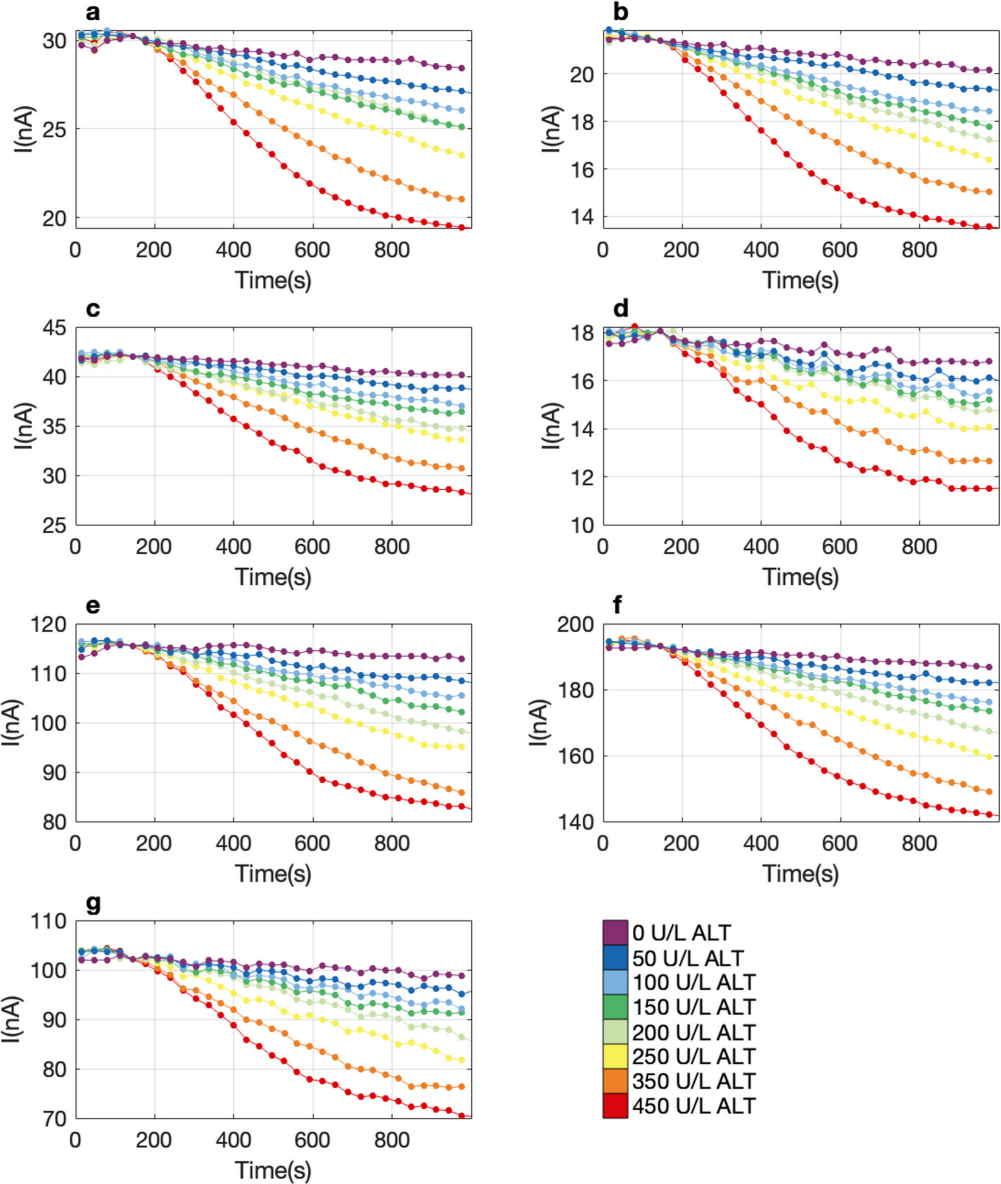
Simultaneous measurement of ALT assay is consistent on seven parallel sensors. **a**–**g** Current (nA) vs time (s) measurement of a dilution series of ALT 0-450 U/L on seven silicon nanowire test sensors on a single sensing die. Each concentration is represented in a different color as indicated in the color bar. Each curve is offset to an equal baseline at 144 (s) The dots on the curves are the measurement cycles where the data was boxcar averaged. The average measurement interval was 31.7 ± 0.12 (s). This figure representative of is a single experiment, and that experiment was repeated three times, and all three were averaged and represented in Fig. 5, subplots (**b**) and (**d**).

The SiNW-FET biosensors used in this study contain an array of 7 nanowire sensors capable of monitoring the consumption of ferricyanide simultaneously. We next sought to compare the response of each nanowire sensor in the array to the ALT assay. A dilution series of ALT samples was delivered to one sensor die and the multiplexed current monitored for 16 min. The consumption of ferricyanide during the assay reaction resulted in a decrease in baseline current with a similar pattern across all the SiNW-FET arrays (Fig. 4). The analogous response of all sensors demonstrates that the ALT assay is robust among SiNW-FET arrays.

### ALT assay data reporting for monitoring DILI

To use SiNW-FET biosensors for the monitoring of DILI, we sought to obtain a single value of enzyme activity from seven simultaneous ALT assay measurements on a SiNW-FET sensing array. Deploying miniaturized sensors in a spatially distributed array increases the confidence of detection by cross-referencing the response from each individual sensor^19,49^. In addition, averaging multiple independent signals improves the signal-to-noise ratio proportional to the square root of the number of signals averaged^50^. Typically, enzyme activity is reported from a fixed time interval throughout a kinetic read. For a near-patient device, the time for the sample to reach the sensor may not be tightly controlled, making it difficult to use a fixed time window from a pre-set point in the reaction for calculation. To address that, we designed an assay reporting method that outputs enzymatic activity independent of assay start time, averaged from seven sensors.

**Fig. 5:**
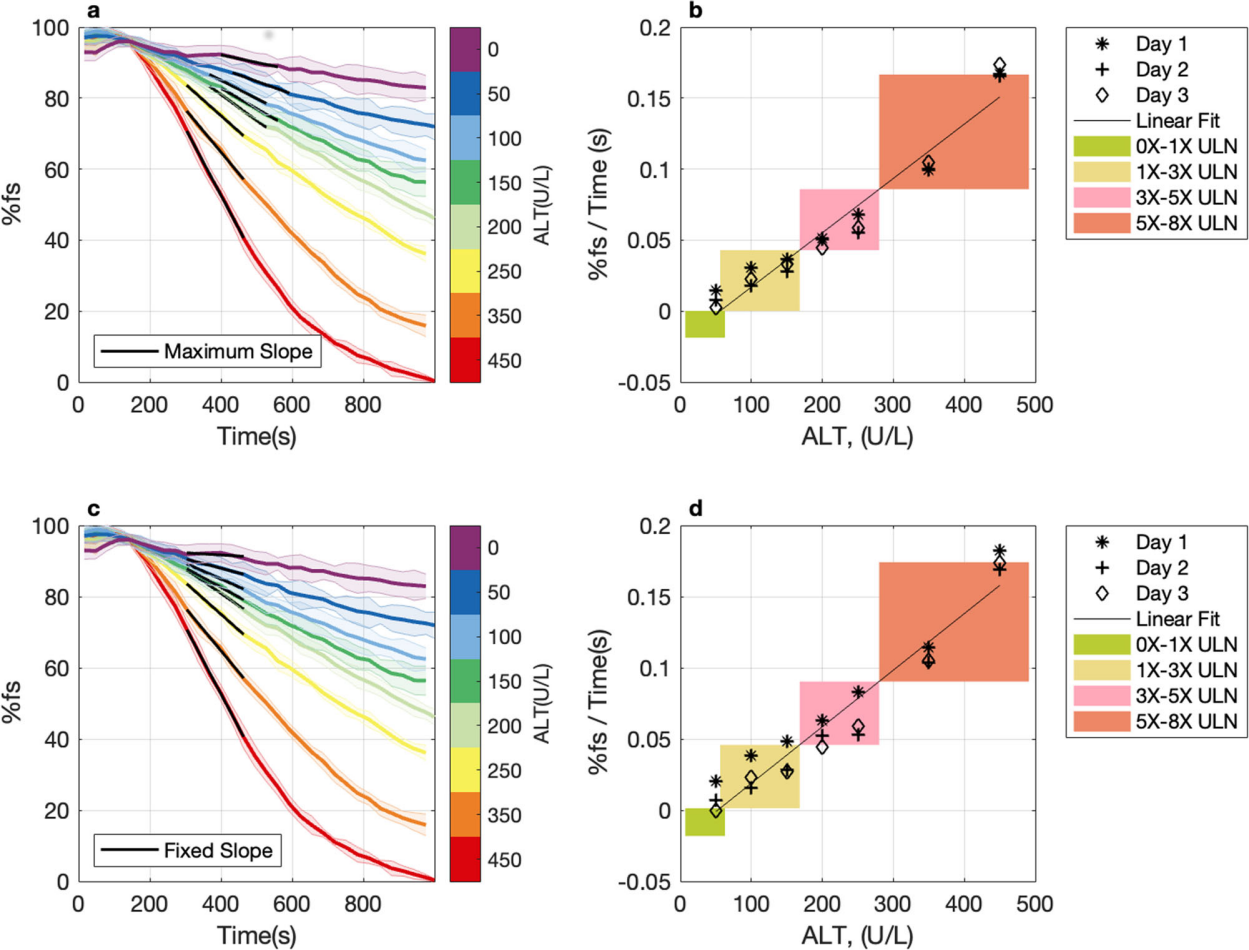
Calculating maximum slope of ALT curves is equivalent to calculating slopes from a fixed interval. **a** Current rescaled to the interval [0 100] vs time bin measurement of a dilution series of ALT 0-450 (U/L). %fs; percent full current scale. Each concentration is represented in a different color as indicated in the color bar, the center line is the mean and the shaded area represents ± standard deviation of *n* = 7 test sensors on a single sensor die. The black line spans the six points of the curve between 300 and 600 (s) with the maximum slope. Each dilution is offset to an equal baseline at 144 (s). The average measurement interval was 31.7 ± 0.12 (s). **b** Maximum slope (%fs / time) vs ALT concentration (U/L). Black line represents linear regression of maximum slope vs ALT concentration. Different shaped markers represent replicate assays collected on separate days. Shaded boxes represent ALT activity levels that correspond to FDA clinical trial stopping rules (Supplementary Table 1). **c** Current rescaled to the interval [0 100] vs time bin measurement of a dilution series of ALT 0-450 (U/L). %fs; percent full current scale. Each concentration is represented in a different color as indicated in the color bar, the center line is the mean and the shaded area represents ± standard deviation of *n* = 7 test sensors on a single sensor die. The black line marks the fixed area of the curve, between 300 and 460 (s), where the slope was calculated. Each dilution is offset to an equal baseline at 144 (s). The average measurement interval was 31.7 ± 0.12 (s). **d** Slope from fixed x-interval 300–460(s) (%fs/Time (s)) vs ALT concentration (U/L). Black line represents linear regression of maximum slope vs ALT concentration. Different shaped markers represent replicate assays collected on separate days. Shaded boxes represent ALT activity levels that correspond to FDA clinical trial stopping rules (Supplementary Table 1).

The current output from individual assays on *n* = 7 SiNW-FET arrays was normalized by the full range of the signal change on each sensor, and ensemble averaged at each time bin (Fig. 5a). The slopes of a linear regression in 160 (s) windows were calculated across the time range of 300–600 (s) and the maximum slope was selected. We constructed a standard curve of maximum slope vs ALT activity from three replicates of an ALT dilution series, collected on three separate days on the same *n* = 7 SiNW-FET arrays on a single sensor die (Fig. 5b). The 95% confidence interval for the slope of the linear regression line was [3.4e−4, 4.3e−4]. To compare the standard curve from the maximum slope to that calculated from a fixed interval, we also calculated the slope of the linear regression line between 300 and 460 (s) (Fig. 5c). We constructed a standard curve containing the slope obtained from 300 to 460 (s) vs ALT activity from the same set of data (Fig. 5d). The 95% confidence interval for the slope of the regression line was [3.5e−4, 4.5e−4]. As the 95% confidence intervals of the slopes from Fig. 5b and d overlap, there is no statistically significant difference between the linear regression lines applied to both datasets. Therefore, taking the maximum slope as the rate of ALT reaction is equivalent to taking the slope from a fixed interval during the reaction. Selecting the maximum slope makes the rate of ALT reaction independent of the exact start time of the reaction, which is highly applicable to a near-patient monitoring device.

### Assessment of interference from endogenous analytes

Lastly, we evaluated five common substances present in whole blood for potential interference in the ALT assay (Supplementary Fig. 4). Assay interference was defined as a statistically significant difference in the slope of the assay calibration line with the interferant from the slope of the assay calibration line with vehicle control. All substances tested showed no significant interference in the ALT assay at above physiological levels, including unconjugated bilirubin (≤3 mg/dL), hemoglobin (≤2.5 g/L), glucose (≤25 mM), pyruvate (≤320 µM), and Intralipid induced lipemia (L-index ≤ 200). The upper limit of normal for the substances tested are as follows, unconjugated bilirubin (0.15 mg/dL), hemoglubin (0.05 g/L), glucose (7.8 mM), and pyruvate (160 µM). While L-index is poorly correlated to serum triglyceride concentration, lipemia is defined as turbidity in serum and plasma. The industry standard assay, Roche Cobas ALTL, has no reported interference up to an L-index of 150. The substances we tested represent a superset of those evaluated for commercially available assays. These results suggest that our assay will perform reliably in a clinically diverse patient population.

## Discussion

In these studies, we have demonstrated the capability of real-time serum ALT activity determination in a clinically relevant range for monitoring DILI using SiNW-FETs. We developed an assay chemistry that couples the ALT-catalyzed production of pyruvate to the reduction of ferricyanide, which can be monitored spectrophotometrically on a microplate reader and electronically on a commercially manufacturable SiNW-FET biosensor array. The assay is linear up to 8X ULN on both the microplate reader and SiNW-FET biosensor array, designed to cover the range required for frequent liver function monitoring in patients at risk for DILI. The measurement results can be obtained within 600 s, providing a rapid and reliable ALT assay reading. Finally, we designed a method for determining the rate of ferricyanide consumption that is independent of assay start time, which will be critically important for future integration of the biosensing platform into a portable, near-patient device.

Many efforts have been made to develop assays with SiNW biosensors, primarily in academic settings^23,51,52^. However, successful commercialization of SiNW-based biosensing technology is scarce due to the limitations in traditional synthesis approaches^51,53^. Notably, we can successfully manufacture the sensor arrays in a cost-efficient wafer-level process, ready for scale-up and close to commercialization. The nanowires in the SiNW-FET biosensor arrays are designed and fabricated with equal width across nanowires, which enables reliable biomarker detection. This is an attribute difficult to obtain using traditional nanowire synthesis methods. Data collection from a single sensor die containing 7 simultaneously recording SiNW-FET arrays on the multiplexing platform improves the signal-to-noise ratio and provides high sensitivity for ALT activity measurement.

A low-cost, portable device for quantitative monitoring of serum ALT activity will relieve the logistical and financial burdens in the way of routine monitoring for liver function and DILI. Colorimetric paper devices for portable ALT measurements have been reported^54,55^, but these devices are marginally quantitative and do not provide a wide enough dynamic range to monitor up to Level 3 DILI, required in pharmaceutical clinical trials. Quantitative measurement of ALT in a portable electronic sensor has been previously described in the literature^37,38,56–61^. Her et al. have demonstrated the possibility of sensing ALT by monitoring pH changes^57^. The three-electrode sensor described by Song et al. produced the lowest LOD of 1.3 U/L^60^ while Thuy and Tseng’s platinum electrode accomplished the largest dynamic range of 10–900 U/L^61^. While these analytical characterizations demonstrate the utility of these assays for ALT quantification, all measurements were carried out in buffer or buffer diluted serum. We describe the first electrical POC assay validated in serum, limiting the practicality of comparisons to these previous characterizations. Furthermore, the current gold standard ALT test is done with an automated analyzer in a centralized laboratory and requires venipuncture blood draw in a clinical setting. Patients with an aversion or fear of needles may delay receiving necessary testing if venipuncture is required^62^. The manufacturable SiNW-FET biosensors described in this paper will be integrated into a platform that requires 10 µl of blood from a finger prick, significantly less than clinical venipuncture and possible in a variety of settings.

In conclusion, we have demonstrated multiplexed electrical detection of serum ALT activity on a close-to-commercialization SiNW-FET biosensor platform. Measurement on multiple sensors simultaneously and subsequent data processing will enable reliable data reporting. We demonstrate the application of an uncoupled SiNW-FET biosensor to kinetically measure the activity of an endogenous human enzyme in undiluted human serum. Integration of the SiNW-FET biosensors described here into a portable platform will provide opportunities for routine monitoring of liver function and can be deployed in many near patient settings.

## Methods

### Reagents

Alanine aminotransferase (ALT) depleted human serum and partially purified human ALT protein were obtained from Aalto Scientific (Eatonton, GA). L-alanine, alpha-ketoglutaric disodium salt dihydrate, potassium ferricyanide, potassium phosphate dibasic, thiamine pyrophosphate, magnesium chloride hexahydrate, 3-aminopropyl-triethoxysilane (APTES), sodium hydroxide, anhydrous ethanol, pyruvate, glucose, unconjugated bilirubin, hemoglobin, and Intralipid (20% emulsion) were obtained from Sigma Aldrich (St. Louis, MO). Pyruvate oxidase was obtained from A.G. Scientific (San Diego, CA). Assay mixture consisted of 500mM L-alanine, 15 mM alpha-ketoglutarate, 3.5 mM potassium ferricyanide, 25 mM potassium phosphate, 0.23 mM thiamine pyrophosphate, 20 mM magnesium chloride and 10 U/mL pyruvate oxidase in ALT depleted human serum.

### Optical detection of ferricyanide reduction

Ferricyanide, a yellow Fe^3+^ complex, is reduced to ferrocyanide, a colorless Fe^2+^ complex. In addition to the electrical measurement described above, this reduction can be monitored spectroscopically at 420 nm. The conversion of ferricyanide to ferrocyanide was measured on a Varioskan LUX^™^ plate reader (Thermo Fisher Scientific, Waltham MA) by monitoring the absorbance of ALT containing assay mixture at 420 ± 6 nm, in 30 s intervals over 1 h at 37 °C. The Varioskan LUX was equipped with a dispenser to inject 196 µl assay mixture to microplate wells containing 4ul ALT enzyme prior to initiating the kinetic read sequence. Data was acquired in SkanIt software version 6.1.1 (Thermo Fisher Scientific, Waltham, MA). Data plotting and calculation of slope from kinetic results was done in MATLAB R2021b (MathWorks, Natick, MA).

### Sensor surface modification

The nanowires were functionalized with primary amines to attract negatively charged species at physiological pH. For this purpose, the sensor die was hydroxylated with oxygen plasma in a Plasma Etch PE-50 (Plasma Etch, Carson City, NV) plasma cleaner at 100 W, 30 cc/min, for 90 s. The sensor was immediately silanized by immersion in 5% APTES in 95% ethanol solution at pH 5.5 for 20 min. The sensors were washed twice in 95% ethanol and once in 0.1 mM NaOH prior to curing overnight in a 120 °C oven.

### Electrical detection of ferricyanide reduction

The silicon nanowire sensor was wire-bonded and mounted on a printed circuit board (PCB) electrically accessible by its finger pads. The device was sealed in a flow chamber together with an Ag/AgCl reference electrode, and solutions containing ALT protein and ALT reaction mixture were hand delivered to the sensor using a syringe. The flow chamber was kept in a dry incubator (Fisher Scientific, Hampton, NH) set to 37 °C for the duration of each experiment. The baseline current of the nanowire decreases as ferricyanide reduces to ferrocyanide due to a change in the charge distribution in the vicinity of the nanowire. The source–drain voltage was 200 mV, the fluid-gate voltage was −400 mV, and the back-gate voltage was 0 V. The source–drain voltage was controlled and monitored using a source meter 2400 (Keithly Instruments, Solon, OH), and both fluid-gate voltage and back-gate voltage were controlled with a lock in amplifier SR810 (Stanford Research Systems, Sunnyvale, CA). Multiplexed measurement of 7 nanowire sensor arrays was achieved by continuously switching the sensor array being monitored by a single source meter. During each cycle, each sensor array is recorded individually, excluding external circuit effects from the measurement.

### Sensor data processing

Sensor data was acquired with custom LabView 2017 software (National Instruments, Austin, TX), stored as ‘.csv’ files and subsequently analyzed using MATLAB R2021b. Measured current obtained from multiple sensors was boxcar averaged at each measurement cycle and offset to an equal baseline at 144 s for each sensor (Fig. 4). The average measurement cycle time was 31.7 ± 0.12 (s). To normalize signals from different sensors with different baselines, the resulting current was rescaled to interval [0 100] by the maximum, *y*_max_, and minimum, *y*_min_ of the data from each sensor

3 $$\% {{{{{\rm{FS}}}}}}=\left(\frac{y-{y}_{{{{{{\rm{min}}}}}}}}{{y}_{{{{{{\rm{max}}}}}}}-{y}_{{{{{{\rm{min}}}}}}}}\right) * 100$$

here %FS represents percent full current scale. The rescaled current values from multiple sensors were subsequently ensemble averaged (Fig. 5a, c). Slope was reported as the maximum slope across a 160 s time window of the curve (Fig. 5b) or from a fixed time interval (Fig. 5d).

### Safety Information

ALT depleted human serum was pre-screened for blood borne pathogens prior to shipment from the manufacturer. Appropriate PPE for working with pre-screened human serum was a lab coat, eye protection, and gloves. Liquid biological waste was treated with 10% bleach prior to disposal in a laboratory sink with copious amounts of water. Plastic consumables that were in contact with biologicals were disposed of by Stericycle (Bannockburn, IL).

### Supplementary information

Supplementary Information

## Data Availability

The data in this manuscript is available upon reasonable request from the corresponding authors, with permission from FemtoDx, Inc.

## Code availability

Essential code in this manuscript is available upon reasonable request from the corresponding authors, with permission from FemtoDx, Inc.
