# Supplementary material for: Alanine aminotransferase assay biosensor platform using silicon nanowire field effect transistors
Source: Commun Eng. 2023 Mar 1;2:8. doi: 10.1038/s44172-023-00057-4 (PMC10956001; doi:10.1038/s44172-023-00057-4)
Supplement: Supplementary file 1 — Supplementary Information [file 44172_2023_57_MOESM1_ESM.pdf]

Supplementary Figures

Supplementary Figure 1

3-Aminopropyltriethoxysilane (APTES) functionalization for ferricyanide and ferrocyanide detection. To compare the signal detection of ferricyanide to ferrocyanide conversion by sensors with or without APTES functionalization, we tested two sensor dies from the same manufacturing batch and with the same patten of design. One sensor die was plasma cleaned with oxygen and silanized by APTES. The other sensor die was untreated. Both dies were tested under the same electrical settings. 100uL of 5mM potassium ferricyanide and 5mM potassium ferrocyanide in phosphate buffered saline (Thermo Fisher, Waltham, MA) were delivered to the sensors in alternating sequence, using a flow chamber. Sensor data was collected with the methods described in the main manuscript.

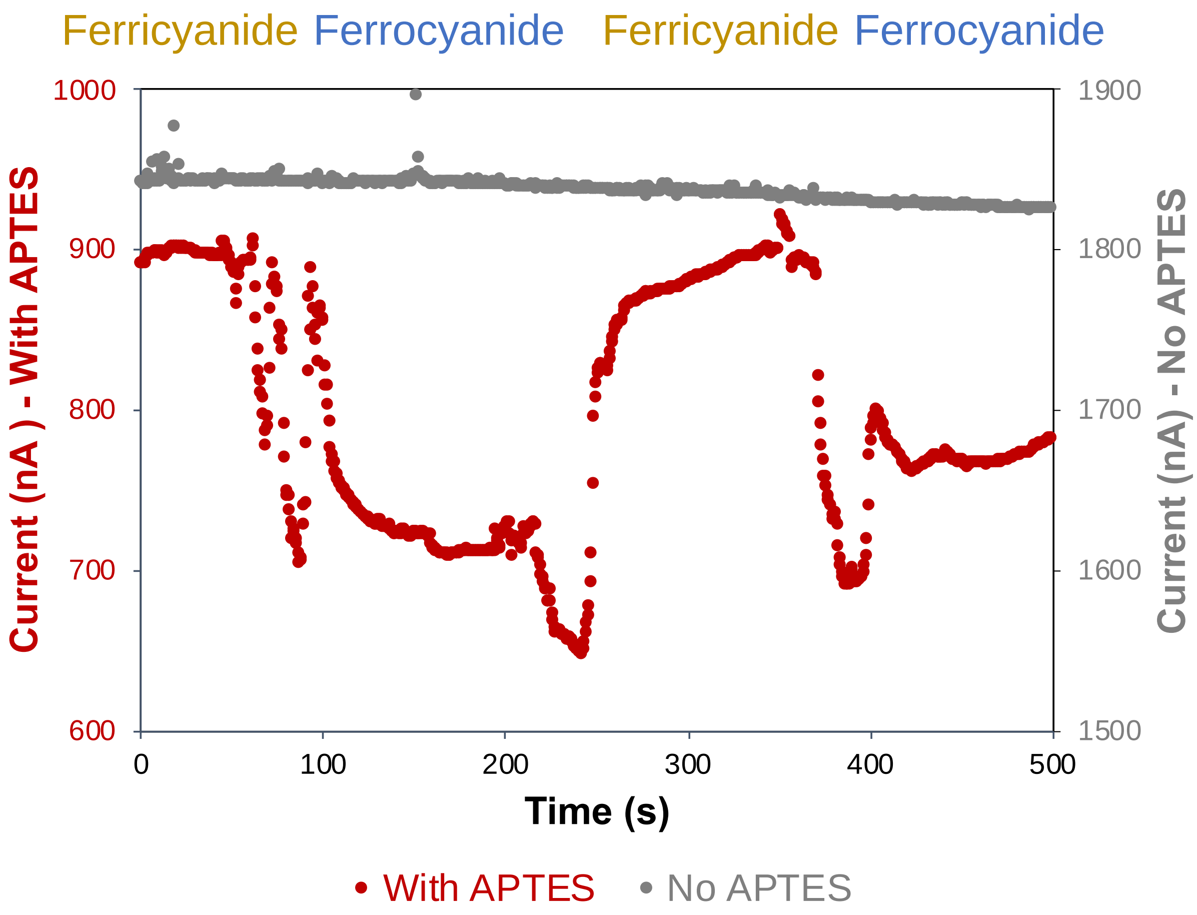

APTES is required to detect the conversion between ferricyanide and ferrocyanide. 5mM potassium ferricyanide and 5mM potassium ferrocyanide were delivered to the sensors in alternating sequence. The sensor without APTES functionalization showed no change between the samples (grey), while APTES functionalized sensor differentiated between ferricyanide and ferrocyanide (red). As explained in the main text, the change in the charge state from ferricyanide to ferrocyanide and the resultant increase in the number of negative charges in the vicinity of the SiNW-FET results in a decrease in measured conductance.

Supplementary Figure 2.

Effect of pH change on current signal in ALT reaction. The measured pH of the reaction mixture in ALT depleted serum prior to adding ALT was 7.23. After adding ALT to the reaction mixture and allowing the reaction to proceed completely, the measured pH of the reaction mixture was 6.99. To evaluate whether the change in current signal observed during the ALT coupled reaction was driven by the observed pH change, we recorded the baseline current on a sensor die for the reaction mixture without ALT at 4 different pH levels. Linear regression was used to calculate the expected change in current for the maximal change in pH of -0.24 observed for the ALT reaction. A full ALT curve was recorded on the same die in pH 6.95 reaction mixture to compare the current change of the full reaction to that of the pH change in reaction mixture. The current signal change of the ALT reaction is -41.30 nA, while the anticipated current change from the linear regression of the pH plot is -1.90 nA. The change in current observed from the ALT reaction is not driven by the pH change of the reaction mixture.

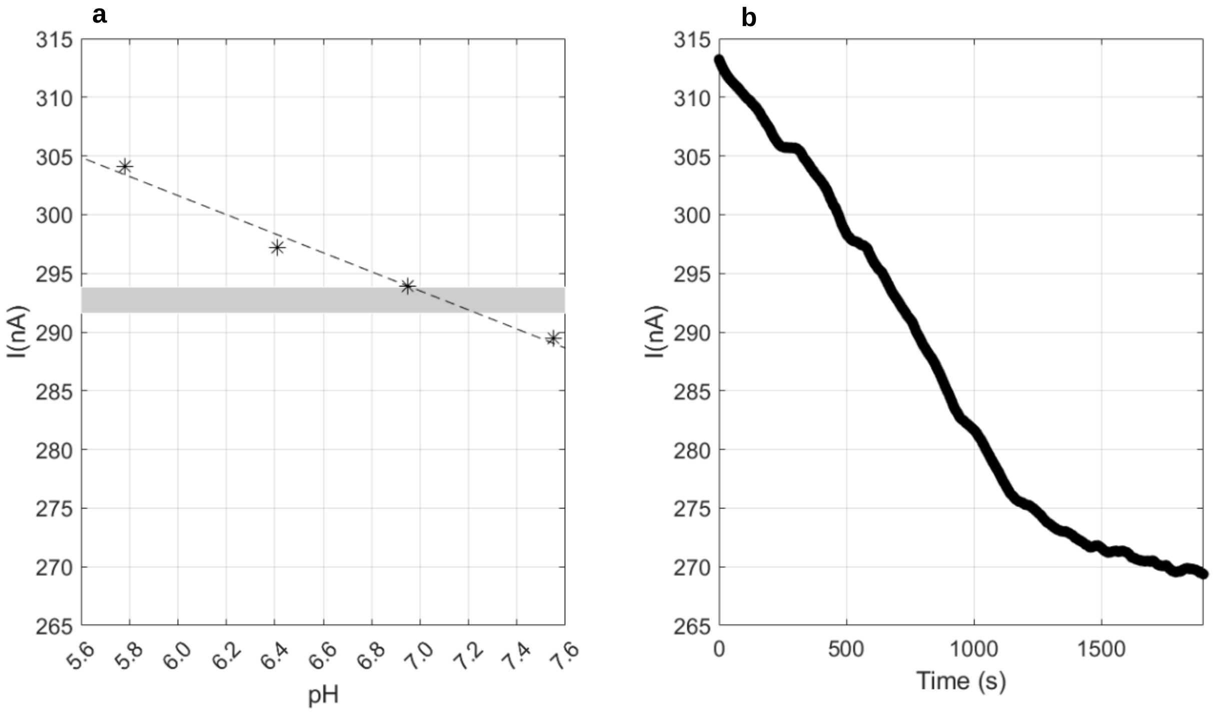

a) Current (nA) vs pH of reaction mixture without ALT. Dotted line represents the linear regression of current vs pH. The 95% confidence interval for the slope was [-11.22 -5.02]. The R^2 of the linear fit was 0.98. The gray rectangle spans the anticipated change in current from the pH change observed in the ALT reaction, from pH 7.23 to pH 6.99**. b)** Current (nA) vs time (s) for the ALT coupled reaction in reaction mixture at pH 6.95.

Supplementary Figure 3.

Assay of ALT Standard. Human ALT, partially purified, was obtained from Aalto Scientific (Eatonton, GA) at a reported concentration of 239,000 U/L. The standard was assayed in our laboratory in accordance with IFCC standards. One unit of ALT is defined as the amount of enzyme producing 1 nmol of pyruvate per minute under IFCC standard conditions: 500mM alanine, 15mM alpha-ketoglutarate, 37C, 100mM Tris pH 7.2.[33] In addition to the IFCC standard components, the enzyme was assayed in the presence of 5mM ferricyanide 0.23mM thiamine pyrophosphate and 10U/L pyruvate oxidase. The reaction was monitored spectrophotometrically at 405nm at 37°C with a Multiskan plate reader (Thermo Fisher, Waltham, MA). Pyruvate 31.3nM-500nM was incubated with the reaction mixture and the absorbance was measured after completion of the reaction at 990 (s). This data was used to calculate the standard curve of pyruvate generated, shown below.

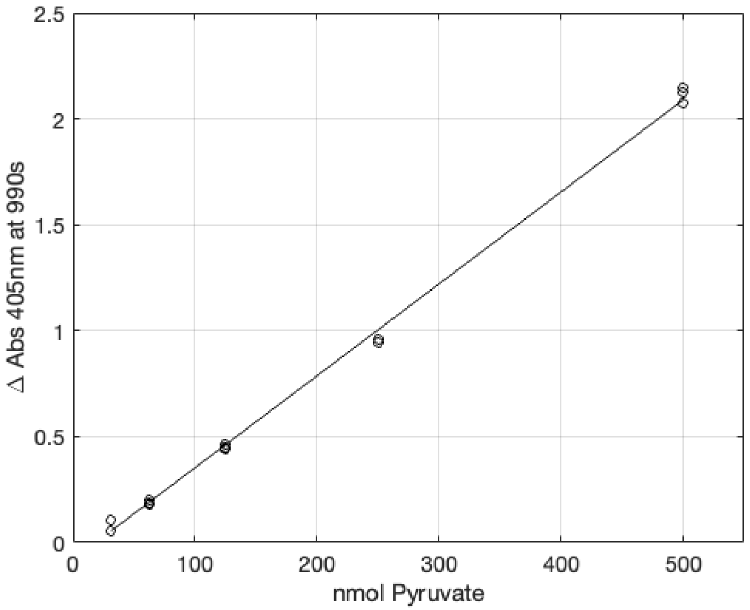

ALT activity (A) was calculated using the following equation:

$$A = \left(\frac{B}{\Delta T * V}\right)$$

Where B is pyruvate generated (nmol), T is reaction time (min), and V is reaction volume (ml). The amount of pyruvate generated (nmol) is calculated from linear regression of the standard curve above. The Human ALT standard was 217,000 U/L when assayed in our laboratory by this method. The enzyme was subsequently aliquoted into single use volumes and stored at -20°C. A single aliquot of enzyme was thawed and used for each of the experiments in the main manuscript.

Supplementary Figure 4.

Evaluation of Potential Interfering Substances

To evaluate the performance of the ALT assay on SiNW FETs in the presence of potential interfering compounds, we spiked in either the interferant or a vehicle control into serum samples for which results of an automated ALT test were available (Cobas Integra 400+ ALTL, Roche Diagnostics USA, Indianapolis, Indiana). ALT samples with interferant or vehicle were delivered to the sensors and the multiplexed current was monitored for 13 minutes. A standard curve for each interferent and its corresponding control were collected on the same day. We defined the criteria for interference as a statistically significant difference between the slopes of the interferant and control calibration curve linear fits. We calculated confidence intervals for each of the slopes of the linear regression line, and slope confidence intervals that overlapped were not considered significantly different.

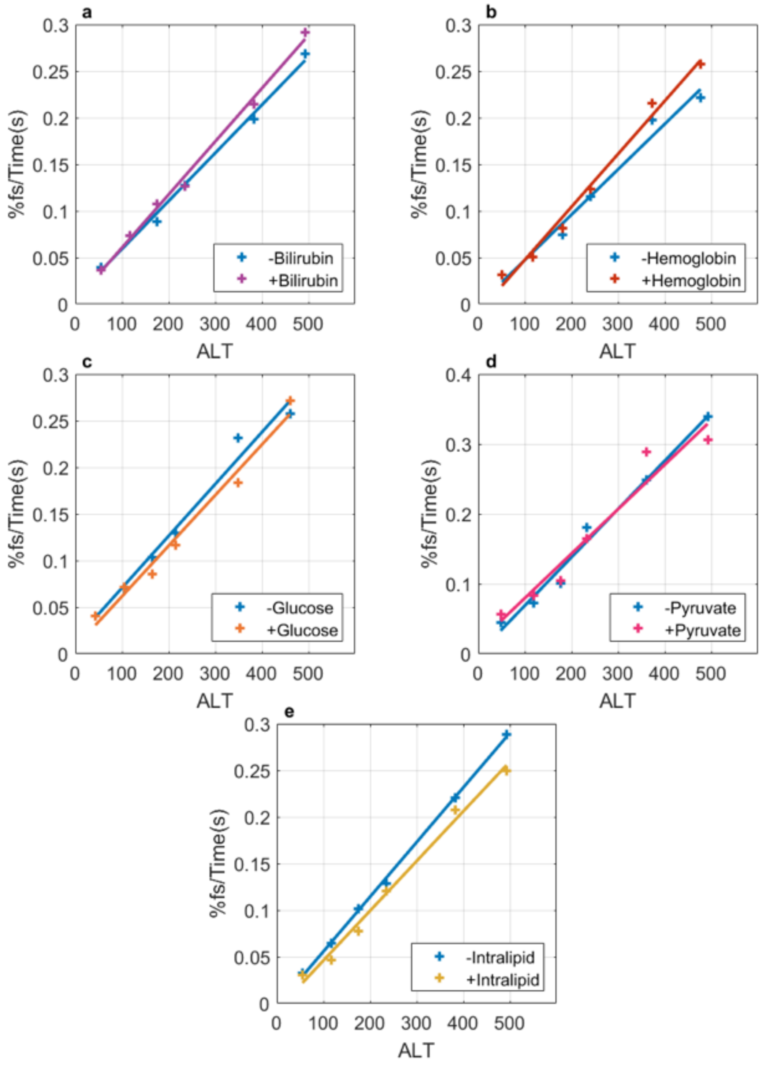

a) Maximum slope (%fs / time) vs ALT concentration (U/L), ± 3 mg/dl bilirubin. Solid lines represent linear regression of maximum slope vs ALT concentration. The 95% confidence intervals for the slope were [5.3e-4, 6.7e-4] for the samples containing bilirubin and [4.7e-4, 6.1e-4] for the control samples. The R^2 of the linear fit was 0.99 for both datasets. **b)** Maximum

slope (%fs / time) vs ALT concentration (U/L), ± 2.5 g/L hemoglobin. Solid lines represent linear regression of maximum slope vs ALT concentration. The 95% confidence intervals for the slopes were [4.8e-4, 6.6e-4] for the samples containing hemoglobin and [3.9e-4, 5.8e-4] for the control samples. The R^2 of the linear fits was 0.99 for the samples containing hemoglobin and 0.98 for the control samples. **c)** Maximum slope (%fs / time) vs ALT concentration (U/L), ± 25mM glucose. Solid lines represent linear regression of maximum slope vs ALT concentration. The 95% confidence intervals for the slope were [4.4e-4, 6.5e-4] for the samples containing glucose and was [4.5e-4, 6.7e-4] for the control samples. The R^2 of the linear fit was 0.98 for both datasets. **d)** Maximum slope (%fs / time) vs ALT concentration (U/L), ± 320 µM pyruvate. Solid lines represent linear regression of maximum slope vs ALT concentration. The 95% confidence intervals for the slope were [4.2e-4, 8.5e-4] for the samples containing pyruvate and was [5.7e-4, 8.1e-4] for the control samples. The R^2 of the linear fits was 0.95 for the samples containing pyruvate and 0.98 for the control samples. **e)** Maximum slope (%fs / time) vs ALT concentration (U/L), ± Intralipid to an L-Index of 200 (Roche Cobas Integra 400+, SI2). Solid lines represent linear regression of maximum slope vs ALT concentration. The 95% confidence intervals for the slopes was [4.7e-4, 5.9e-4] for the samples containing Intralipid and was [5.5e-4, 6.0e-4] for the control samples. The R^2 of the linear fit was 0.99 for both datasets.

Supplementary Tables

Supplementary Table 1.

United States Food and Drug Administration Clinical Trial Stopping Rules

**ALT Level**	**Description**
1X ULN	ULN at beginning of trial
3X ULN	Withdraw if symptoms are present
5X ULN	Withdraw after two weeks
8X ULN	Withdraw immediately

<u>Reference for Supplementary Table 1</u>

Guidance for Industry Drug-Induced Liver Injury: Premarketing Clinical Evaluation. U.S. Food and Drug Administration. Silver Spring, MD. (2009).
